# Importance of Anatomic Peculiarities and Ultrasound-Guided Electromyography of the Extensor Indicis Proprius Muscle

**DOI:** 10.7759/cureus.20154

**Published:** 2021-12-04

**Authors:** Fatma Elleuch, Wafa Elleuch, Baya M Kallel, Houcem Harbi, Sameh Ghroubi, Habib M Elleuch

**Affiliations:** 1 Physiology, Habib Bourguiba Hospital, University of Sfax, Sfax, TUN; 2 Physical Medicine & Rehabilitation, Habib Bourguiba Hospital, University of Sfax, Sfax, TUN; 3 General Surgery, Research Laboratory LR21ES04, Habib Bourguiba Hospital, University of Sfax, Sfax, TUN; 4 Physical Medicine & Rehabilitation, Research Laboratory LR20ES09, Habib Bourguiba Hospital, University of Sfax, Sfax, TUN

**Keywords:** residents, electromyography, ultrasound, anatomy, extensor indicis proprius muscle

## Abstract

The extensor indicis proprius muscle (EIPM) is considered a key muscle in the assessment of the level of the neurologic lesion causing any motor or sensory medio-cubital impairment of the hand.

The aim of this study is to illustrate the anatomical peculiarities of the EIPM, the ultrasound (US) anatomy of the inferoposterior part of the forearm, and the technique of US-guided electromyography (EMG) of the EIPM.

The US-guided EMG of the EIPM is technically easy and safe for young practitioners, provided there is a good knowledge of US anatomy of the inferoposterior part of the forearm.

## Introduction

Nowadays, residents and young practitioners still have practical difficulties in electromyographic detection of certain small and deep muscles, such as the extensor indicis proprius muscle (EIPM). They need to acquire some procedural skills for electromyography (EMG) needle placement. For this, the musculoskeletal ultrasound (US) would be of considerable and very precious help. Indeed, it facilitates the identification of these difficult muscles and provides a good knowledge of the forearm ultrasound anatomy [1].

This manuscript aims to illustrate the anatomical peculiarities of the EIPM, the US anatomy of the inferoposterior part of the forearm, and the technique of US-guided EMG of the EIPM. The latter is considered a key muscle in the assessment of the level of a neurologic lesion causing any motor or sensory medial/cubital impairment of the hand [2].

## Technical report

**Figure 1:**
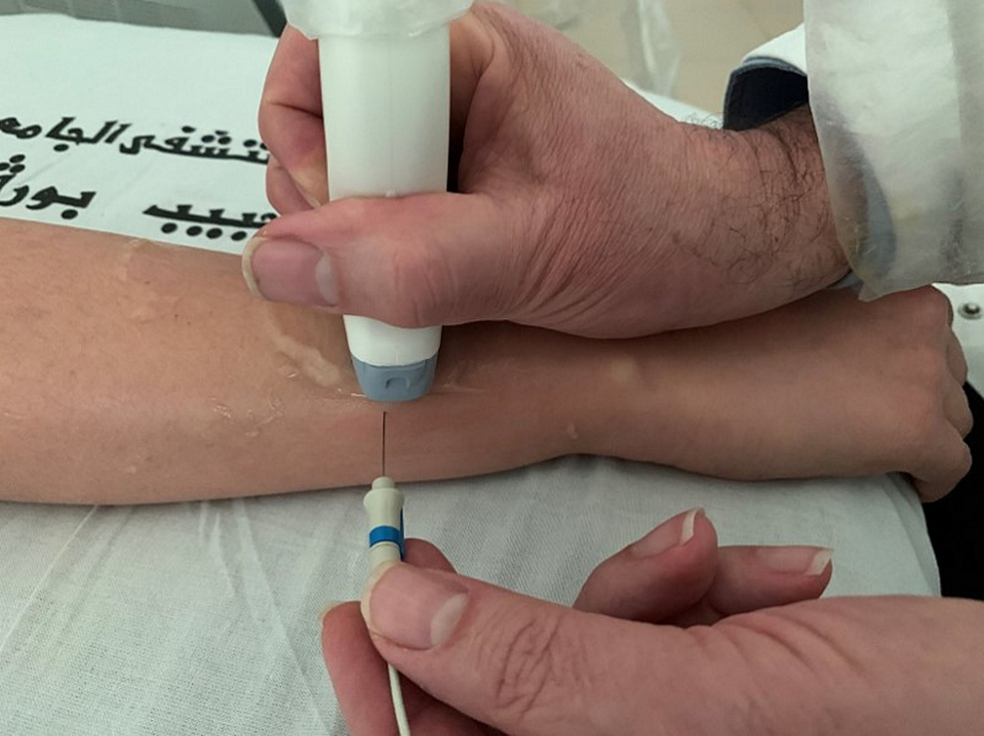
Performing the ultrasound of the forearm The ultrasound probe is placed at the level of the distal part of the posteromedial side of the forearm, i.e., 4 to 5 cm above the wrist. The electromyography (EMG) needle is then inserted laterally and perpendicular to the medial edge of the forearm. Note: This individual depicted in this figure was a resident who provided consent for demonstration purposes.

**Figure 2:**
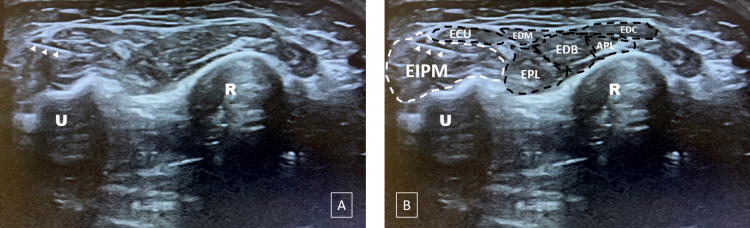
Ultrasound image (A) Ultrasound section in the distal part of the forearm without and (B) with delineation of muscle structures. The extensor indicis proprius muscle (EIPM) is located at the deep medial side of the posterior compartment of the forearm. This ultrasound section shows also the electromyography (EMG) needle (arrowheads) in the EIPM. APL: abductor pollicis longus; ECU: extensor carpi ulnaris; EDB: extensor digitorum brevis; EDC: extensor digitorum communis; EDM: extensor digitorum mini; EIP: extensor indicis proprius; EPL: extensor pollicis longus; R: radius; U: ulna Note: These images were taken from a voluntary medical resident.

**Figure 3:**
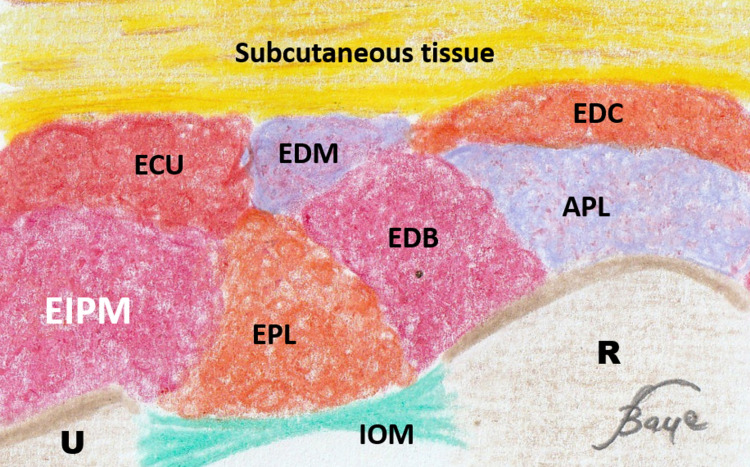
Schematic view Schematic and anatomical illustration of the ultrasound section of Figure 2. APL: abductor pollicis longus; ECU: extensor carpi ulnaris; EDB: extensor digitorum brevis; EDC: extensor digitorum communis; EDM: extensor digitorum mini; EIP: extensor indicis proprius; EPL: extensor pollicis longus; R: radius; U: ulna

A good knowledge of the ultrasound anatomy of the forearm allows locating the EIPM easily to prick it with an EMG needle. First, the practitioner must place the ultrasound probe at the level of the distal part of the posteromedial side of the forearm (Figure 1). The US image should then show all muscles of the posterior compartment of the forearm and, in particular, the EIPM on the extreme medial side as shown in Figure 2. It is then easy and safe to prick the EIPM with the EMG needle. The physician should also know that mobilization of the index is mandatory for the exact US localization of this muscle. Figure 3 shows a schematic and anatomical illustration of this US picture.

## Discussion

Anatomy reminder

The EIPM is a thin muscle of the posterior compartment of the forearm. It originates from the distal two-thirds of the posterolateral side of the ulna. Then, it follows a downward path on the medial side of the extensor digitorum longus. At the dorsal side of the carpal bones, the EIPM narrows into a tendon that courses obliquely down to the metacarpal level where it joins the ulnar side of the extensor digitorum tendon. This union forms the extensor apparatus of the index finger [3].

The main function of this muscle is the extension of the index finger by acting at the metacarpophalangeal and interphalangeal joints. In addition, the EIPM may incidentally provoke a weak wrist extension as it crosses this joint.

The main anatomic particularity of the EIPM is that it is the only muscle of the forearm posterior compartment to be innervated by radial nerve branches strictly issued from the C8-T1 roots.

US contribution for performing needle EMG of the EIPM

Two recent studies have shown the significant contribution of musculoskeletal US in teaching residents and young electromyographers to perform needle EMG of the EIPM [1, 4]. Moreover, these studies also showed that trainees have high rates of failure of standard EMG detection of the EIPM.

US also has an important role in detecting EIPM anatomical variants that can be bifidity with a double insertion on the index finger or by giving one tendon to the index and another to the middle finger or thumb. Exceptionally, EIPM can give three slips for the index or one for the index, one for the ring finger, and a third one for the middle [5-6].

Diagnostic value of the extensor indicis proprius muscle

The EIPM electromyographic study is essential for determining the level of non-traumatic nerve damage at the origin of any motor or sensory medial/cubital impairment as in the case of non-traumatic hand amyotrophy.

Moreover, thanks to the fact that it is the only muscle to be innervated strictly by the C8-T1 branches, the EIPM is a key muscle. Thus, the electromyographic study allows distinguishing between a medial/cubital truncal lesion and a proximal C8-T1 root impairment. Indeed, it is the only muscle whose neurogenic impairment leads to the diagnosis of a neurologic lesion at the level of the primary lower trunk instead of a lesion of the anteromedial secondary trunk [7].

In general, the clinical signs of this type of nerve impairment are 1) atrophy of the hand (thenar and hypothenar eminences and interosseous muscles), 2) paraesthesia of the finger(s), and 3) muscle testing revealing the weakness of hand muscles. For example, a patient can have muscle testing at 3 (range: 0 - 5) of the abductor pollicis brevis and the EIPM [8].

## Conclusions

Thanks to its anatomical peculiarities, the EIPM is a key muscle in determining the level of the neurologic lesion that causes motor or sensory medial/cubital impairment of the hand. Ultrasound guidance greatly facilitates the electromyographic exploration of the EIPM (a thin and deep muscle), particularly for trainees and young practitioners who have to be familiar with the anatomy of the inferoposterior part of the forearm.
